# Nitrogen-fixing populations of Planctomycetes and Proteobacteria are abundant in surface ocean metagenomes

**DOI:** 10.1038/s41564-018-0176-9

**Published:** 2018-06-11

**Authors:** Tom O. Delmont, Christopher Quince, Alon Shaiber, Özcan C. Esen, Sonny TM Lee, Michael S. Rappé, Sandra L. McLellan, Sebastian Lücker, A. Murat Eren

**Affiliations:** 1 Department of Medicine, University of Chicago, Chicago, IL USA; 2 Warwick Medical School, University of Warwick, Coventry, UK; 3 Graduate Program in the Biophysical Sciences, University of Chicago, Chicago, IL USA; 4 Hawaii Institute of Marine Biology, University of Hawaii at Manoa, Kaneohe, HI USA; 5 School of Freshwater Sciences, University of Wisconsin-Milwaukee, Milwaukee, WI USA; 6 Department of Microbiology, Radboud University, Nijmegen, The Netherlands; 7 Josephine Bay Paul Center, Marine Biological Laboratory, Woods Hole, MA USA; 8 Committee on Microbiology, University of Chicago, Chicago, IL USA

**Keywords:** Microbial ecology, Metagenomics, Marine microbiology, Bacterial genomics

## Abstract

Nitrogen fixation in the surface ocean impacts global marine nitrogen bioavailability and thus microbial primary productivity. Until now, cyanobacterial populations have been viewed as the main suppliers of bioavailable nitrogen in this habitat. Although PCR amplicon surveys targeting the nitrogenase reductase gene have revealed the existence of diverse non-cyanobacterial diazotrophic populations, subsequent quantitative PCR surveys suggest that they generally occur in low abundance. Here, we use state-of-the-art metagenomic assembly and binning strategies to recover nearly one thousand non-redundant microbial population genomes from the TARA Oceans metagenomes. Among these, we provide the first genomic evidence for non-cyanobacterial diazotrophs inhabiting surface waters of the open ocean, which correspond to lineages within the Proteobacteria and, most strikingly, the Planctomycetes. Members of the latter phylum are prevalent in aquatic systems, but have never been linked to nitrogen fixation previously. Moreover, using genome-wide quantitative read recruitment, we demonstrate that the discovered diazotrophs were not only widespread but also remarkably abundant (up to 0.3% of metagenomic reads for a single population) in both the Pacific Ocean and the Atlantic Ocean northwest. Our results extend decades of PCR-based gene surveys, and substantiate the importance of heterotrophic bacteria in the fixation of nitrogen in the surface ocean.

## Abstract (web summary)

Recovery of population genomes from surface ocean samples identified non-cyanobacterial diazotrophs that were widespread and abundant, including Proteobacteria and Planctomycetes, indicating their importance for nitrogen fixation in this environment.

## Main

Marine microbial communities play a critical role in biogeochemical fluxes and regulating climate^1–3^, but their activity in the euphotic zone of low latitude oceans is often limited by the availability of inorganic fixed nitrogen^4,5^. Thus, biological fixation of gaseous dinitrogen in the surface ocean is a globally important process that contributes to the ocean’s productivity and can potentially enhance the sequestration of carbon through the biological pump^6,7^. Microbial populations that can fix nitrogen (termed diazotrophs) encompass a wide range of archaeal and bacterial lineages^8,9^. However, diazotrophs within the bacterial phylum Cyanobacteria, in particular, are considered to be responsible for a substantial portion of nitrogen input in the surface ocean^10–12^. Studies employing cultivation and flow cytometry^13–17^ have characterized multiple cyanobacterial diazotrophs and shed light on their functional lifestyles^18–20^. PCR amplicon surveys of the nitrogenase reductase *nifH* gene have indicated that the ability to fix nitrogen is also found in bacterial lineages that include the phyla Proteobacteria, Firmicutes and Spirochaetes^9,21,22^, suggesting the presence of heterotrophic bacterial diazotrophs (HBDs) that contribute to the introduction of fixed nitrogen in the surface ocean. Quantitative surveys of non-cyanobacterial *nifH* genes have indicated that HBDs are diverse and active, but relatively rare in the surface ocean^23–29^, and efforts to access genomic representatives through cultivation and culture-independent techniques have so far only been successful in coastal waters^30,31^, limiting our understanding of their ecophysiology in the open ocean.

Here, we have used metagenomic assembly, binning and curation strategies to create a non-redundant database of archaeal, bacterial and eukaryotic genomes from the TARA Oceans project^32^. We characterized nearly one thousand microbial genomes from the surface samples of four oceans and two seas, revealing nitrogen-fixing populations within the phylum Proteobacteria, as well as in the Planctomycetes, which is a widespread phylum^33^ that has never been linked to nitrogen fixation previously. These discoveries enable the genome-wide tracking of these populations, through which we determined that putative HBDs are orders of magnitude more abundant in surface seawater across large regions of the global open ocean compared to previous estimates that relied on PCR amplifications.

## Results

The 93 TARA Oceans metagenomes we analysed correspond to a size fraction targeting free-living microorganisms (0.2–3 μm) from 61 surface samples and 32 samples from the deep chlorophyll maximum layer of the water column (Supplementary Table 1). Presumed absent from this size fraction are the majority of those bacterial and archaeal cells that have a symbiotic relationship with eukaryotes, form large aggregates or attach to large particles. Of 33.7 billion metagenomic reads, 30.9 billion passed quality control criteria and were used as input for 12 metagenomic co-assemblies (1.14–5.33 billion reads per set) using geographically bounded samples (Supplementary Fig. 1). A total of 42,193,607 genes were identified in scaffolds longer than 1,000 nucleotides (see Supplementary Table 2 for a summary of the assembly statistics). A combination of automatic and manual binning was applied to each co-assembly output, which resulted in 957 manually curated, non-redundant metagenome-assembled genomes (MAGs) containing 2,288,202 genes (Supplementary Fig. 1; also see ref. ^34^ for an automatic binning effort that includes larger size fractions).

Our MAGs belonged to the domains Bacteria (*n* = 820), Eukarya (*n* = 72) and Archaea (*n* = 65) (Supplementary Table 3), and recruited 2.11 billion quality controlled reads (6.84% of the data set) when we mapped the metagenomic data back to this collection. The genomic completion estimates for archaeal and bacterial MAGs based on domain-specific single-copy core genes averaged to 79% and 76.1%, respectively, and resolved to the phyla Proteobacteria (*n* = 432), Bacteroidetes (*n* = 113), Euryarchaeota (*n* = 65), Verrucomicrobia (*n* = 65), Planctomycetes (*n* = 43), Actinobacteria (*n* = 37), Chloroflexi (*n* = 34), *Candidatus* Marinimicrobia (*n* = 27), Acidobacteria (*n* = 6), Cyanobacteria (*n* = 6), Spirochaetes (*n* = 5), Firmicutes (*n* = 2), Ignavibacteriae (*n* = 1) and diverse members of the Candidate Phyla Radiation (*n* = 4). We could assign only 6.33% of the bacterial and archaeal MAGs to described genera. Eukaryotic MAGs were substantially larger than bacterial and archaeal MAGs (7.24 Mbp versus 2.26 Mbp and 1.47 Mbp on average, respectively) and were dominated by a small number of genera: *Micromonas* (*n* = 14), *Emilliana* (*n* = 14), *Bathycoccus* (*n* = 8) and *Ostreococcus* (*n* = 4). Recovery of these MAGs complements decades of cultivation efforts by providing genomic context for lineages missing in culture collections (for example, Euryarchaeota and *Candidatus* Marinimicrobia), and allowed us to search for diazotrophs within a large pool of marine microbial populations.

### Genomic stability of a well-studied nitrogen-fixing symbiotic population at large scale

Our genomic collection included six cyanobacterial MAGs, one of which (ASW 00003) contained genes that encode the catalytic (*nifHDK*) and biosynthetic (*nifENB*) proteins required for nitrogen fixation^8^. This MAG, which we recovered from the Atlantic southwest metagenomic co-assembly, showed remarkable similarity to the genome of the symbiotic cyanobacterium ‘*Candidatus* Atelocyanobacterium thalassa’^35,36^ (previously known as UCYN-A) sorted by flow cytometry from the North Pacific gyre (GenBank accession no. CP001842.1). Besides their comparable size of 1.43 Mbp (MAG ASW 00003) and 1.46 Mbp (consensus genome from isolated cells), their average nucleotide identity was 99.96% over the 1.43 Mbp alignment. ‘*Ca*. A. thalassa’ is a diazotrophic taxon that lacks key metabolic pathways and lives in symbiosis with photosynthetic eukaryotic cells^19,36^. The high genomic similarity between ASW 00003 and the ‘*Ca*. A. thalassa’ genome sorted by flow cytometry demonstrates the accuracy of our metagenomic workflow.

### Genomic evidence for nitrogen fixation by Proteobacteria and Planctomycetes

Besides the cyanobacterial MAG, we also identified seven Proteobacteria and two Planctomycetes MAGs in our collection that contained the complete set of genes for nitrogen fixation. To the best of our knowledge, these MAGs (HBD-01 to HBD-09) represent the first genomic evidence of putative HBDs inhabiting the surface of the open ocean (Table 1). They were obtained from the Pacific Ocean (*n* = 6), Atlantic Ocean (*n* = 2) and Indian Ocean (*n* = 1), and possessed relatively large genomes (up to 6 Mbp and 5,390 genes) and a GC content ranging from 50% to 58.7%. One of the Proteobacterial MAGs resolved to the genus *Desulfovibrio* (HBD-01). The remaining MAGs from this phylum correspond to lineages within the orders Desulfobacterales (HBD-02), Oceanospirillales (HBD-03, HBD-04, HBD-05) and Pseudomonadales (HBD-06, HBD-07) (Table 1). The phylogenetic assignment of one Planctomycetes MAG (HBD-08) with a low completion estimate (33.5%) could not be resolved beyond the phylum level, possibly due to missing phylogenetic marker genes for taxonomic inferences. However, the length of this MAG (4.03 Mbp) suggests that its completion may have been underestimated, as we have observed in previous studies^37,38^. The second Planctomycetes MAG (HBD-09) was affiliated with the family Planctomycetaceae (order Planctomycetales) based on its single-copy core genes. This MAG contained a large fragment of the 16S rRNA gene (1,188 nt; Supplementary Table 4) for which the best match to any characterized bacterium in the NCBI’s non-redundant database was *Algisphaera agarilytica* (strain 06SJR6-2, NR_125472) with 88% identity.

**Table 1:** Summary of the genomic features of HBDs

Population	Status	Region	Length (Mbp)	N50	No. of contigs	GC (%)	C/R (%)	Taxonomy
HBD-01	Reference	PSW	3.67	48,153	118	52.56	97.7/4.4	Proteobacteria (genus *Desulfovibrio*)
HBD-02	Reference	PSW	6.00	20,964	405	53.07	97.1/5.9	Proteobacteria (family Desulfobacteraceae)
HBD-03	Reference	ION	4.47	57,949	110	52.39	97.5/8.1	Proteobacteria (family Oceanospirillaceae)
HBD-04	Reference	PON	4.29	48,897	138	52.41	89.7/6.1	Proteobacteria (family Oceanospirillaceae)
HBD-05	Reference	PSE	4.15	65,098	94	53.27	47.7/5.8	Proteobacteria (family Oceanospirillaceae)
HBD-06	Reference	ANW	5.49	76,792	112	54.23	98.1/5.6	Proteobacteria (order Pseudomonadales)
	Redundant	PON	5.56	65,956	134	53.69	86.8/5.6	Proteobacteria (order Pseudomonadales)
	Redundant	PSW	5.33	101,765	128	54.14	98.3/8.7	Proteobacteria (order Pseudomonadales)
	Redundant	PSE	5.29	51,046	226	54.46	98.3/7.4	Proteobacteria (order Pseudomonadales)
HBD-07	Reference	ANW	3.99	10,488	487	58.72	91.2/4.3	Proteobacteria (order Pseudomonadales)
	Redundant	ANE	3.14	5,704	610	58.71	66.5/1.8	Proteobacteria (order Pseudomonadales)
HBD-08	Reference	PSW	4.03	10,413	480	52.57	33.5/6.0	Planctomycetes
HBD-09	Reference	PSW	5.86	79,495	113	49.98	97.3/4.6	Planctomycetes (family Planctomycetaceae)
	Redundant	PSE	5.68	10,913	655	49.98	83.0/4.7	Planctomycetes (family Planctomycetaceae)

For each HBD population, the status column differentiates MAGs that were included as reference in our non-redundant genomic collection from the ones that were also recovered from other geographic regions. Regions of recovery include ANW (Atlantic northwest), ANE (Atlantic northeast), ION (Indian Ocean north), PON (Pacific Ocean north), PSE (Pacific Ocean southeast) and PSW (Pacific Ocean southwest). The column ‘C/R’ displays the completion and redundancy estimates for each MAG. The phylum-level taxonomy, as well as the lowest taxonomic for which the MAG was assigned below phyla, is displayed in the column ‘Taxonomy’.

**Fig. 1:**
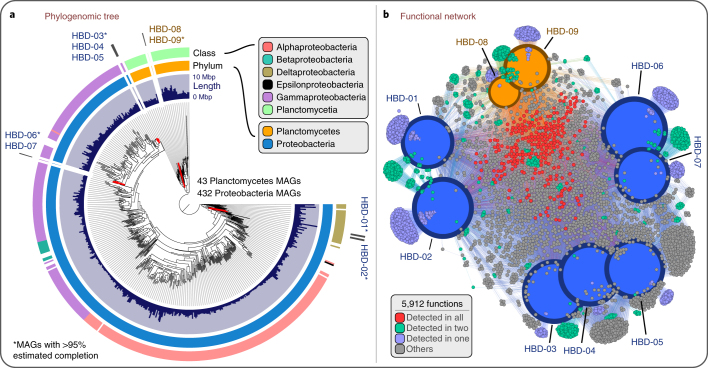
Nexus between phylogeny and function of HBDs. **a**, Phylogenomic analysis of 432 Proteobacteria MAGs and 43 Planctomycetes MAGs in the non-redundant genomic database (including the nine HBDs) using a collection of 37 phylogenetic marker gene families. Layers surrounding the phylogenomic tree indicate genome size and taxonomy of each MAG at the phylum and class level. **b**, Functional network of the nine HBDs based on a total of 5,912 identified gene functions. Size and colour of genomic nodes represent the number of detected functions and MAG taxonomy, respectively. Colours of functional nodes indicate their occurrence in the different HBDs.

We placed the nine HBDs in a phylogenomic analysis of the 432 Proteobacteria and 43 Planctomycetes MAGs using a set of 37 marker gene families (Fig. 1a; for an interactive version see https://anvi-server.org/merenlab/tara_hbds). The two deltaproteobacterial HBDs were closely related to each other, but not adjacent in the phylogenomic tree. The HBDs within Oceanospirillales (*n* = 3), Pseudomonadales (*n* = 2) and Planctomycetes (*n* = 2) formed three distinct phylogenomic lineages. These results suggest that closely related populations of diazotrophs inhabit the surface ocean, and nitrogen fixation genes occur sporadically among diverse putatively heterotrophic marine microbial lineages, consistent with previous investigations^39^.

Our initial binning results included 120 redundant MAGs that were observed multiple times in independent co-assemblies (Supplementary Table 5). Although they are not present in our final collection of 957 non-redundant MAGs (for an accurate assessment of the relative abundance of microbial populations), we used this redundancy to investigate the stability of the phylogeny and functional potential of populations recovered from multiple geographical regions. For instance, we characterized the genomic content of HBD-06 from the Atlantic northwest (5.49 Mbp) and from each of the three Pacific Ocean regions (5.56, 5.33 and 5.29 Mbp in regions PON, PSW and PSE, respectively) (Table 1 and Supplementary Table 5). Average nucleotide identities between the Atlantic MAG and three Pacific MAGs ranged from 99.89% to 99.97% over more than 97% of the genome length. We observed similar trends for HBD-07 and HBD-09 (Table 1 and Supplementary Table 5). The complete set of nitrogen fixation genes was present in all of the redundant MAGs, demonstrating the large-scale stability of this functional trait in these HBDs.

On average, the proportion of genes of unknown function was 27.6% (±2.63%) for the proteobacterial HBDs and 49.3% (±0.5%) for the Planctomycetes HBDs, reflecting our greater lack of functional understanding of the latter taxonomic group of diazotrophs. The 37,582 total genes identified in the nine HBDs encoded for 5,912 known functions (Supplementary Table 6), and a network analysis of HBDs based on known functions organized them into four distinct groups corresponding to Deltaproteobacteria, Oceanospirillales, Pseudomonadales and Planctomycetes (Fig. 1b), mirroring the results of our phylogenomic analysis. A large number of the functions identified in these HBDs (4,224 out of 5,912) were unique to one of the four groups (Fig. 1b and Supplementary Table 6). The relatively weak overlap of known functions between these groups indicates that the ability to fix nitrogen in marine populations may not be associated with a tightly defined functional lifestyle. The HBDs we characterized appeared to be involved in different steps of the nitrogen cycle (for example, denitrification for HBD-06) and possessed distinct strategies regulating nitrogen fixation (see section ‘Functional differences between HBDs’ in the Supplementary Information for additional functional insights), but shared traits related to energy conservation, motility, nutrient acquisition and gene regulatory processes. Swimming motility, which has previously been suggested as a potential mechanism to find anaerobic microniches favourable to nitrogen fixation^28,40^, was a common trait we observed in all the HBDs and may be an indication of particle-attached lifestyle rather than the symbiotic lifestyles observed in some cyanobacterial diazotrophs.

### The taxonomy of HBDs is coherent with the phylogeny of nitrogen fixation genes

**Fig. 2:**
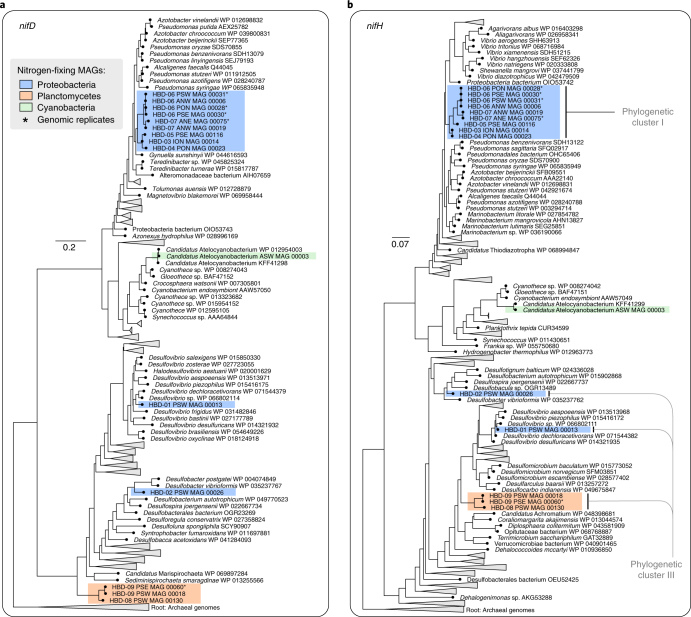
Phylogeny of nitrogen fixation genes. **a**,**b**, Phylogenetic analysis of *nifD* (**a**) and *nifH* (**b**) occurring in the 15 nitrogen-fixing MAGs (including five redundant MAGs and ‘*Ca*. A. talassum’) we identified from TARA Oceans in relation to 252 and 316 reference proteins, respectively. MAGs are coloured based on their phylogenetic affiliation at the phylum level.

Our phylogenetic analysis of the catalytic *nifH* and *nifD* genes from a wide range of diazotrophs placed our HBDs in four distinct lineages (Fig. 2). Also included in this analysis were the genomic replicates that were removed from the non-redundant genomic collection. These replicates clustered with their representative MAGs in the phylogenetic tree, revealing near-identical nitrogen fixation genes in geographically distant HBDs. HBD-01 (*Desulfovibrio*) and HBD-02 (Desulfobacterales) were clustered with close taxonomic relatives. In addition, the gammaproteobacterial HBDs were most closely related to reference genomes of the genera *Pseudomonas* and *Azotobacter* from the same class. Finally, the *nifD* and *nifH* genes we identified in the Planctomycetes HBDs formed distinct clusters, which was particularly apparent for *nifD* (Fig. 2). All of the catalytic and biosynthetic genes for nitrogen fixation were located in a single operon in the Planctomycetes HBD-09 genome (HBD-08 was too fragmented to determine their organization). The agreement between the taxonomy of HBDs and their placement in the functional gene-based phylogeny, along with the synteny of genes involved in nitrogen fixation (see Supplementary Information), both favour a scenario where transmission of these genes is mainly vertical in the surface ocean, contributing to the ongoing debate regarding the extent of horizontal transmission for this key functionality^9,30,41^.

### HBDs are not only diverse but are also abundant in the surface ocean

**Fig. 3:**
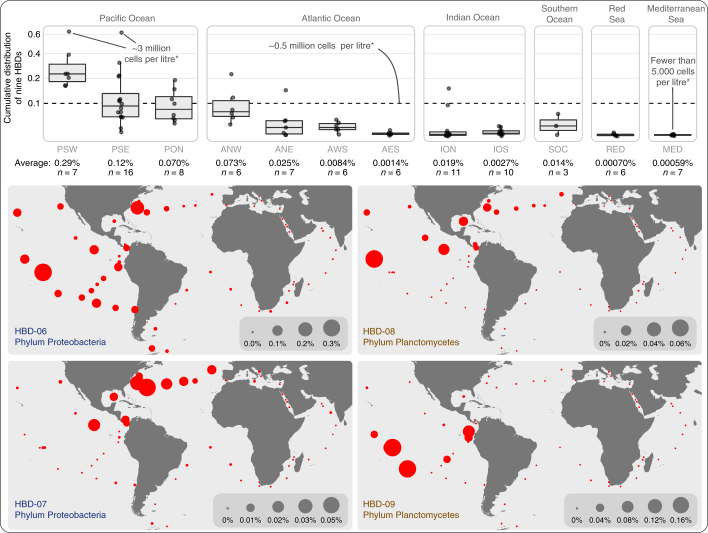
Abundance of nitrogen-fixing populations of Planctomycetes and Proteobacteria in the surface ocean. Top: boxplots display the square-root-normalized cumulative relative distribution of the Planctomycetes (*n* = 2) and Proteobacteria (*n* = 7) HBDs in 93 metagenomes corresponding to 12 marine geographic regions (*assuming that each litre in the surface ocean contains 0.5 billion archaeal and bacterial cells^83^). Boxes represent the first quartile, median and third quartile of distribution values, and whiskers of 1.5 × interquartile range. Bottom: maps show the niche partitioning of HBD-06, HBD-07, HBD-08 and HBD-09 at the surface of four oceans and two seas (61 metagenomes from surface samples).

The cumulative relative abundance of the Planctomycetes and Proteobacteria HBDs in the metagenomic data set averaged 0.01% and 0.05%, respectively. In particular, HBD-06, the diazotrophic population that recruited the largest number of reads with an average and maximum relative abundance of 0.025% and 0.33% across all metagenomes, ranked 47th in our database of 957 MAGs (Supplementary Table 3). The relative abundance of Proteobacteria and Planctomycetes HBDs was very low in the Mediterranean Sea and Red Sea (0.00064% on average). In contrast, they were substantially enriched in metagenomes from the Pacific Ocean (0.14% on average) compared to the other regions (Fig. 3). In fact, the Pacific Ocean metagenomes contained 81.4% of the 17.8 million reads that were recruited by the HBD MAGs from the entire metagenomic data set. In particular, the two most abundant Proteobacteria and Planctomycetes HBDs (HBD-06 and HBD-09) showed a broad distribution (Fig. 3) and were significantly enriched in this ocean (Welch’s test, *P* < 0.005). HBD-06 was also abundant in the northwest region of the Atlantic Ocean and to a lesser extent in the Southern Ocean, revealing that the ecological niche of a single HBD population can encompass multiple oceans and a wide range of temperatures (Supplementary Table 3). Interestingly, HBD-07 and HBD-08, which are phylogenetically and functionally closely related to HBD-06 and HBD-09, respectively, were not only less abundant, but also exhibited a different geographical distribution (Fig. 3). We could not explain the increased signal for the nine HBDs in a few geographic regions using temperature, salinity or the concentration of essential inorganic chemicals including oxygen, phosphate and nitrate (Supplementary Table 1).

To reconcile the abundance of nitrogen-fixing populations in the surface of the open ocean with the inclusion of HBDs described in this study, we used the previous PCR-based estimations of the abundance of non-cyanobacterial *nifH* gene phylotypes. Quantitative PCR (qPCR) surveys have estimated that non-cyanobacterial *nifH* gene phylotypes generally range from 10 to 1,000 copies, and rarely reach 0.1 million copies per litre^23–27,42^. We translated genome-wide quantitative read recruitment of our HBDs into cells per litre (see Supplementary Information for details). Our estimates suggest that the nine populations of HBDs characterized in this study collectively correspond to 0.72 million cells per litre on average (and up to 3.16 million cells) in the surface of the Pacific Ocean, and 0.077 million cells per litre in the other regions. HBD-06 alone might contribute about 0.31 million cells per litre in the Pacific Ocean. These results indicate that HBD populations are orders of magnitude more abundant than previously thought in metagenomes covering large regions of the surface ocean.

### PCR assays confirm the occurrence of Planctomycetes *nifH* genes in the surface ocean

We tracked HBDs at the long-term field study of Station ALOHA (22° 45′ N, 158° 00′ W) in the oligotrophic North Pacific Subtropical Gyre to compare the sensitivity of metagenomics and PCR surveys. The nine HBDs were below the detection limit in a data set of 624.2 million metagenomic reads originating from Station ALOHA^43^, indicating that HBDs are not as abundant at this location as they are in other regions of the Pacific Ocean (Supplementary Table 7). We developed digital droplet (dd)PCR assays for the two Planctomycetes *nifH* genes, and could detect HBD-08 at ~750 copies per litre in samples from Station ALOHA^44^ (Supplementary Table 7). We could also detect HBD-09 at levels near the limit of detection, confirming the occurrence of Planctomycetes *nifH* genes in the surface ocean.

### Reconstructed *nifH* genes are more abundant than previously characterized *nifH* genes in surface ocean metagenomes

**Fig. 4:**
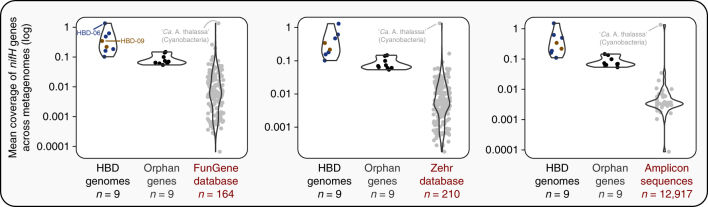
Relative abundance of the TARA Oceans *nifH* genes in the context of reference collections and amplicons. Violin plots summarizing the average mean coverage of *nifH* genes retrieved in this study, *nifH* reference databases^46,47^ and *nifH* amplicon sequences from a large-scale survey^39^ across 93 TARA Oceans metagenomes using a competitive read recruitment strategy. The 18 *nifH* genes retrieved in this study were separated into two groups (‘HBD genomes’ and ‘Orphan genes’ for which we only have a scaffold) and compared to a database of *nifH* gene sequences. For each gene sequence, the coverage values were corrected by excluding nucleotide positions with coverage in the 1st and 4th quartiles to minimize the effect of non-specific mapping.

The non-redundant collection of 957 curated MAGs in which we searched for HBDs encompassed only 5.42% of the genes in our metagenomic assembly outputs. To identify more *nifH* genes, we also investigated those occurring in the remaining ‘orphan’ scaffolds (39,510,139 genes). Our search based on amino acid similarity with the HBD database resulted in the recovery of nine additional non-redundant *nifH* genes (Fig. 4 and Supplementary Table 8). Eight of them originated from the Pacific Ocean metagenomic co-assemblies, substantiating the unequal distribution patterns for nitrogen fixation genes we observed at the MAG level (Fig. 3). Phylogenetic analysis on these *nifH* genes affiliated them with Elusimicrobia (*n* = 2), Firmicutes (*n* = 2), Proteobacteria (*n* = 1), Spirochaeta (*n* = 1), Verrucomicrobia (*n* = 1), a group of uncultured bacteria (*n* = 1), and Euryarchaeota (*n* = 1) (Supplementary Fig. 1). This primer-independent survey identified a wide range of *nifH* gene lineages that spanned all four of the previously described phylogenetic clusters^45^ (Supplementary Table 8). The average nucleotide identity of short metagenomic reads each *nifH* gene recruited was between 97.4% and 100%, and above 99% for each of the nine HBDs (Supplementary Table 8), suggesting that these *nifH* gene sequences correspond to highly homogeneous phylotypes. Despite their high abundance in the surface ocean, most of these *nifH* genes were not in the NCBI non-redundant database, or reference *nifH* collections^46,47^, and none of them occurred in a large-scale amplicon survey of the surface ocean^39^, even when considering the subtle variations these phylotypes maintain in the environment (Supplementary Table 8). Our in silico analysis of widely used primer sequences (see Methods) revealed mismatches to these *nifH* genes, which is a likely reason for this discrepancy (Supplementary Table 8).

We used previously characterized reference and amplicon *nifH* gene sequences to recruit reads from metagenomes to estimate their relative abundance (see Methods). The large majority of these sequences were undetected in the TARA Oceans metagenomes (Supplementary Table 9), and the few sequences recruiting reads were less abundant than the *nifH* genes we reconstructed, confirming the remarkable abundance of the HBDs we characterized (Fig. 4). A notable exception was the ‘*Ca*. A. thalassa’ sequence, which was also present in our MAG database. Finally, the number of reads orphan *nifH* genes recruited suggests that HBDs abundant in the surface ocean might not be limited to Planctomycetes and Proteobacteria (Fig. 4).

### A binomial naming for the HBDs characterized from multiple geographic regions

Most of the MAGs we characterized in our study correspond to unknown genera, but the lack of cultured representatives prevents a formal taxonomic characterization of these lineages. Here we suggest tentative names for the HBDs we independently characterized from multiple geographic regions (that is, those for which we have genomic replicates) using the candidatus status and binomial naming system: ‘*Candidatus* Azoaequarella praevalens’ gen. nov., sp. nov. (HBD-06) and ‘*Ca*. Azopseudomonas oceani’ gen. nov., sp. nov. (HBD-07) within the order Pseudomonadales (unknown family), and ‘*Ca*. Azoplanctomyces absconditus’ gen. nov., sp. nov. (HBD-09) within the phylum Planctomycetes (unknown order and family).

## Discussion

The nine HBDs we describe in this study represent the first genomic insights into nitrogen-fixing surface ocean populations that are not affiliated with Cyanobacteria, and their high-resolution niche partitioning through genome-wide read recruitment. These HBDs include two Planctomycetes populations, which is the first observation of diazotrophy in this phylum. Seawater samples analysed from Station ALOHA in the Pacific Ocean substantiated the presence of Planctomycetes *nifH* genes using metagenomic-guided ddPCR. These findings complement decades of PCR amplicon surveys, and corroborate the relevance of metagenomic assembly and binning strategies to improve our understanding of microbial communities inhabiting the largest biome on Earth. For instance, HBDs were mostly enriched in regions of the Pacific Ocean where iron bioavailability is known to be a limiting factor for cyanobacterial diazotrophs^7^. Iron bioavailability is required for nitrogen fixation but is also particularly important for photosynthesis^48^. Thus, marine systems co-limited by nitrogen and iron may represent appropriate ecological niches for HBDs, where they could be the main sources of inorganic fixed nitrogen input into the surface ocean.

Our study reveals that populations of HBDs within Proteobacteria and Planctomycetes, as well as putative diazotrophs within other archaeal and bacterial phyla, can be abundant in the surface ocean, occasionally across wide ecological niches spanning a large range of temperatures. Our investigation takes advantage of unprecedented amount of shotgun metagenomic sequencing data to investigate the diversity of *nifH* genes without primer bias, which led to the identification of a mismatch in nifH4, a widely used degenerate PCR primer targeting the *nifH* gene^39,49,50^. Although these findings substantiate the previous observations made through PCR amplicon surveys regarding the diversity of HBDs in the surface of the ocean^23–27,39^, they also demonstrate that amplicon surveys may have underestimated the abundance of HBDs by multiple orders of magnitude, and provide a potential explanation for the paradox between high nitrogen fixation rates in the Pacific Ocean and the low abundance of diazotrophs previously estimated in this region^50^. Overall, our investigation emphasizes the need to reassess the role of HBDs in oceanic primary production. As their contribution to the nitrogen cycle has yet to be demonstrated, additional environmental surveys, transcriptomic analyses and cultivation efforts will be essential to establish the lifestyles of HBDs in the open ocean, and to determine the mechanisms and environmental conditions supporting nitrogen fixation in the water column.

## Methods

The URL https://merenlab.org/data/2017_Delmont_et_al_HBDs/ contains a reproducible workflow that extends the descriptions and parameters of the programs used here for (1) metagenomic binning, (2) identification and curation of MAGs, (3) identification of Candidate Phyla Radiation MAGs and (4) profiling of MAGs and *nifH* genes in the entire metagenomic data set.

### TARA Oceans metagenomes

We acquired 93 metagenomes from the European Bioinformatics Institute (EBI) repository under project ID ERP001736, and quality filtered the reads using the illumina-utils library^51^ v1.4.1 (available from https://github.com/meren/illumina-utils). Noisy sequences were removed using the program ‘iu-filter-quality-minoche’ with default parameters, which implements a noise filtering as described in ref. ^52^. Supplementary Table 1 reports accession numbers and additional information (including the number of reads and environmental metadata) for each metagenome.

### Metagenomic co-assemblies, gene calling and binning

We organized the data set into 12 ‘metagenomic sets’ based on the geographic coordinates of metagenomes (Supplementary Table 1). We co-assembled reads from each metagenomic set using MEGAHIT^53^ v1.0.3, with a minimum scaffold length of 1 kbp, and simplified the scaffold header names in the resulting assembly outputs using anvi’o^38^ v2.3.0 (available from https://merenlab.org/software/anvio). For each metagenomic set, we then binned scaffolds >2.5 kbp (>5 kbp for the Southern Ocean) following the workflow outlined in ref. ^38^. Briefly, (1) anvi’o was used to profile the scaffolds using Prodigal^54^ v2.6.3 with default parameters to identify genes (Supplementary Table 2), and HMMER^55^ v3.1b2 to identify genes matching to archaeal^56^ and bacterial^57–60^ single-copy core gene collections; (2) Centrifuge^61^ was used with NCBI's NT database to infer the taxonomy of genes (as described in https://merenlab.org/2016/06/18/importing-taxonomy); (3) short reads were mapped from the metagenomic set to the scaffolds using Bowtie2^62^ v2.0.5 and the recruited reads stored as BAM files using samtools^63^; (4) anvi’o was used to profile each BAM file to estimate the coverage and detection statistics of each scaffold, and to combine mapping profiles into a merged profile database for each metagenomic set. We then clustered scaffolds with the automatic binning algorithm CONCOCT^57^ by constraining the number of clusters per metagenomic set to 100 to minimize the ‘fragmentation error’ (when multiple clusters describe one population), with the exception of the Southern Ocean (25 clusters) and the Pacific Ocean southeast (150 clusters) metagenomic sets. Finally, we manually binned each CONCOCT cluster (*n* = 1,175) using the anvi’o interactive interface. Supplementary Table 10 reports the genomic features (including completion and redundancy values) of the characterized bins.

### Identification and curation of MAGs

We defined all bins with >70% completeness or >2 Mbp in length as MAGs (Supplementary Table 2). We then individually refined each MAG as outlined in ref. ^64^, and renamed scaffolds they contained accordingly to their MAG ID to ensure that the names of all scaffolds in MAGs we characterized from the 12 metagenomic sets were unique.

### Taxonomic and functional inference of MAGs

We used CheckM^65^ to infer the taxonomy of MAGs based on the proximity of 43 single-copy gene markers within a reference genomic tree. We also used Centrifuge, RAST^66^ and manual BLAST searches of single-copy core genes against the NCBI's non-redundant database to manually refine the CheckM taxonomic inferences, especially regarding the archaeal and eukaryotic MAGs. We also used the occurrence of bacterial single-copy core genes to identify MAGs affiliated to the Candidate Phyla Radiation (as described in https://merenlab.org/2016/04/17/predicting-CPR-Genomes/). Supplementary Table 4 reports our curated taxonomic inference of MAGs. We used KEGG (the 14 April 2014 release) to identify functions and pathways in MAGs. We also used RAST to identify functions in 15 MAGs that contained the complete set of nitrogen fixation genes (originally identified from the KEGG pathways). Supplementary Tables 6 and 11 report the RAST and KEEG results, respectively. We used Gephi^67^ v0.8.2 to generate a functional network using the Force Atlas 2 algorithm to connect MAGs and RAST functions. Node sizes were correlated to the number of edges they contained, which resulted in larger nodes for MAGs compared to functions.

### Characterization of a non-redundant database of MAGs

We concatenated all scaffolds from the genomic database of MAGs into a single FASTA file and used Bowtie2 and samtools to recruit and store reads from the 93 metagenomes. We used anvi’o to determine the coverage values, detection and relative distribution of MAGs and individual genes across metagenomes (Supplementary Table 12). The Pearson correlation coefficient of each pair of MAGs was calculated based on their relative distribution across the 93 metagenomes using the function ‘cor’ in R^68^ (Supplementary Table 5). Finally, NUCmer^69^ was used to determine the average nucleotide identity (ANI) of each pair of MAGs affiliated to the same phylum for improved performance (the Proteobacteria MAGs were further split at the class level) (Supplementary Table 5). MAGs were considered redundant when their ANI reached 99% (minimum alignment of >75% of the smaller genome in each comparison) and the Pearson correlation coefficient was above 0.9. We then selected a single MAG to represent a group of redundant MAGs based on the largest ‘completion minus redundancy’ value from single-copy core genes for Archaea and Bacteria, or longer genomic length for Eukarya. This analysis provided a non-redundant genomic database of MAGs. We performed a final mapping of all metagenomes to calculate the mean coverage and detection of these MAGs (Supplementary Table 3 reproducible workflow).

### Statistical analyses

STAMP^70^ and Welch’s test were used to identify non-redundant MAGs that were significantly enriched in the Pacific Ocean compared to all the other regions combined. Supplementary Table 3 reports the *P* values for each MAG.

### World maps

We used the ggplot2^71^ package for R to visualize the metagenomic sets and relative distribution of MAGs in the world map.

### Phylogenomic analysis of MAGs

We used PhyloSift^72^ v1.0.1 with default parameters to infer associations between MAGs in a phylogenomic context. Briefly, PhyloSift (1) identifies a set of 37 marker gene families in each genome, (2) concatenates the alignment of each marker gene family across genomes, and (3) computes a phylogenomic tree from the concatenated alignment using FastTree^73^ v2.1. We rooted the phylogenomic tree to the phylum Planctomycetes with FigTree^74^ v1.4.3, and used anvi’o to visualize it with additional data layers.

### Binomial naming of HBDs

The following is a brief explanation of the binomial naming of three populations of HBDs we characterized from multiple geographic regions:

*Azoaequarella praevalens* (N.L. n. azotum [from Fr. n. azote (from Gr. prep. a, not; Gr. n. zôê, life; N.Gr. n. azôê, not sustaining life)], nitrogen; N.L. pref. azo-, pertaining to nitrogen; L. v. aequare, to equalize; N.L. fem. n. Azoaequarella, the nitrogen equalizer; L. part. adj. praevalens, very powerful, very strong, here prevalent).

*Azopseudomonas oceani* (N.L. n. azotum [from Fr. n. azote (from Gr. prep. a, not; Gr. n. zôê, life; N.Gr. n. azôê, not sustaining life)], nitrogen; N.L. pref. azo-, pertaining to nitrogen; Gr. adj. pseudês, false; Gr. fem. n. monas, a unit, monad; N.L. fem. n. Azopseudomonas, nitrogen-fixing false monad; L. gen. n. oceani, of the ocean).

*Azoplanctomyces absconditus* (N.L. n. azotum [from Fr. n. azote (from Gr. prep. a, not; Gr. n. zôê, life; N.Gr. n. azôê, not sustaining life)], nitrogen; N.L. pref. azo-, pertaining to nitrogen; Gr. adj. planktos, wandering, floating; Gr. masc. n. mukês, fungus; N.L. masc. n. Azoplanctomyces, nitrogen-fixing floating fungus; L. part. adj. absconditus, hidden).

### Identification of additional *nifH* sequences in orphan scaffolds

DIAMOND^75^ was used to generate a database of *nifH* genes we identified in the nine HBDs, and to search for additional *nifH* amino acid sequences within the genes Prodigal identified in scaffolds longer than 1,000 nucleotides. We considered only hits with an e-value of <1e-50, and defined them as *nifH* genes only when (1) ‘nitrogenase’ was the top blastx hit against the NCBI’s nr database, and (2) the characteristic [4Fe-4S]-binding site (Prosite signature PDOC00580) was present in their amino acid sequence.

### Variation of metagenomic reads the *nifH* genes recruit

We concatenated all *nifH* genes (orphan genes, as well as those in HBDs) into a single FASTA file. To study their variation in the environment, we used this FASTA file to recruit reads from all metagenomes, and profiled the resulting mapping results with anvi’o as described in the section ‘Metagenomic co-assemblies, gene calling and binning’. We created an anvi’o collection linking each gene to a unique bin ID, and then used the program ‘anvi-get-short-reads-from-bam’ to extract from the BAM files metagenomic reads each *nifH* gene recruited. Finally, we used blastn to estimate the average nucleotide identity of metagenomic reads to the *nifH* genes. Supplementary Table 8 reports the search results.

### Affiliating *nifH* genes with predetermined phylogenetic clusters

We affiliated the TARA Oceans *nifH* genes with predetermined phylogenetic clusters and subclusters using a classification and regression tree method^76^.

### Searching *nifH* genes in existing sequence databases and amplicons

We searched TARA Oceans *nifH* genes in three databases, and a large amplicon survey. These databases included (1) ‘NCBI nr’, NCBI non-redundant database, (2) the ‘FunGene database’, *nifH* genes curated from NCBI GenBank database and stored in the FunGene database^46^ (available from https://fungene.cme.msu.edu/), and (3) the ‘Zehr Database’, a *nifH* gene repository curated from the NCBI GenBank database and maintained by the Zehr Laboratory^47^ (June 2017 release, available from https://www.jzehrlab.com). We also used the amplicon sequences from a large-scale survey of the *nifH* genes in the surface ocean using nested degenerate primers^39^. To search for our sequences in these resources, we used blastn^77^ with default parameters and only considered matches with a minimum alignment length of 100 nt.

### Identifying mismatches between *nifH* genes and degenerate primers

We created a program (see Code availability) to determine all sequence combinations of 12 commonly used degenerate primers and compare them to the TARA Oceans *nifH* genes to assess their compatibility.

### Mean coverage of *nifH* gene sequences from reference collections and amplicons

We included the 18 non-redundant *nifH* genes we recovered in our study in each of the three non-redundant reference collections: the FunGene database (genes that were not affiliated with *nifH* based on their functional annotation were removed), the ‘Zehr Database’, and the *nifH* amplicon sequences from ref. ^39^. We then used CD-HIT^78^ with a 99% sequence similarity cutoff to independently remove redundancy in these three collections. To estimate the mean coverage of all nucleotide sequences from these three non-redundant collections, we recruited reads from all metagenomes and profiled the resulting mapping results with anvi’o as described in the section ‘Metagenomic co-assemblies, gene calling and binning’. For the analysis of the amplicon sequences, we used blastn to search amplicon sequences that recruited any read from the metagenomes in the FunGene database (with a minimum alignment of 100 nt) to identify those that correspond to *nifH*. We then used blast to combine all *nifH* amplicons that match to the *nifH* gene of ‘*Ca*. A. thalassa’, and combined all matches into a single unit corresponding to this population. We used the R package ggplot2^71^ to display the interquartile range of the mean coverage of *nifH* genes across metagenomes as violin plots, and finalized this figure and others using the open-source vector graphics editor Inkscape (https://inkscape.org/).

### Phylogenetic analysis of *nifD* and *nifH* genes

We built a database using the amino acid sequences of *nifD* and *nifH* genes identified in this study, as well as the protein reference sequences for *nifD* and *nifH* genes we identified in the NCBI’s non-redundant database, and imported it into ARB v.5.5-org-9167^79^. In ARB, we aligned sequences to each other using ClustalW^80^, manually refined alignments, and calculated phylogenetic trees with PhyML^81^ using the ‘WAG’ amino acid substitution model, and a 10% conservation filter.

### Quantification using ddPCR analysis of *nifH* genes

We designed primers specifically targeting the two Planctomycetes population *nifH* genes using primer3^82^ (Supplementary Table 7), and analysed samples from the ALOHA station in the Pacific Ocean^44^ with ddPCR on a Bio-Rad QX200 Droplet Digital PCR system in a reaction volume of 20 μl following the protocols of the manufacturer (Bio-Rad Laboratories). The samples were also tested using the previously described primers for the ϒ-24774A11 target^27^. Artificial constructs of each expected amplicon served as positive controls. We verified ddPCR results for the HBD-09 target using endpoint PCR employing forward and reverse primers and gel visualization (if sample material was available).

### Code availability

The URL https://merenlab.org/data/2017_Delmont_et_al_HBDs serves a reproducible bioinformatics workflow, and https://goo.gl/fZPvWw serves the ad hoc program to identify mismatches between *nifH* genes assembled from metagenomes and commonly used degenerate primers.

### Data availability

All data our study used or generated are publicly available. Accession ID ERP001736 serves TARA Oceans metagenomes through the European Bioinformatics Institute. We stored scaffolds of >2.5 kbp generated from the 12 metagenomic co-assemblies in NCBI Bioproject PRJNA326480. We have also made publicly available the raw assembly results that include scaffolds > 1 kbp (10.6084/m9.figshare.4902920), amino acid sequences for 42.2 million genes identified in raw assembly results (10.6084/m9.figshare.4902917), the FASTA files for our final collection of 957 non-redundant MAGs (10.6084/m9.figshare.4902923), the anvi’o summary of non-redundant MAGs and their distribution across metagenomes (10.6084/m9.figshare.4902926), the self-contained anvi’o split profiles for each non-redundant MAG (10.6084/m9.figshare.4902941), short reads our *nifH* genes recruited from TARA Oceans metagenomes along with their identity statistics to the consensus gene sequence (10.6084/m9.figshare.5259424), and the redundant and non-redundant versions of the FunGene database, Zehr database and *nifH* amplicon sequences we used in our study (10.6084/m9.figshare.5259421).

## Supplementary information

Supplementary Information Supplementary Notes, Supplementary References, Supplementary Figures 1 and 2, Supplementary Table References.

Reporting Summary

Supplementary Table 1 Summary of the 93 metagenomes from TARA Oceans, and the 12 geographic regions they represent.

Supplementary Table 2 Summary of the co-assembly and binning outputs for each metagenomic set.

Supplementary Table 3 Genomic features of 957 MAGs from the non-redundant genomic database. A two-sided *t*-test was performed to compare the relative distribution of each MAG in the Pacific Ocean compared to all other locales.

Supplementary Table 4 The 16S rRNA gene sequence identified in HBD-09.

Supplementary Table 5 Genomic features, Pearson correlation (based on the relative distribution in 93 metagenomes) and average nucleotide identity of 1,077 MAGs from the redundant genomic database.

Supplementary Table 6 RAST subsystems and KEGG modules for the nine HBDs.

Supplementary Table 7 Digital droplet PCR assays targeting the nifH genes of HBD08 and HBD-09 (phylum Planctomycetes) in DNA samples from Station ALOHA in the Pacific Ocean. The table lists the newly designed primers and summarizes detection levels in copies per litre.

Supplementary Table 8 Main characteristics of 18 *nifH* genes retrieved in this study, the similarity of reads they recruited across 93 TARA Oceans metagenomes, best matches against the NCBI non-redundant database, *nifH* reference databases and amplicon sequences from a largescale survey, and compatibility with commonly used PCR primers. The table lists nucleotide sequences of the TARA Oceans *nifH* genes found in MAGs and orphan scaffolds, and mean coverage of the TARA Oceans *nifH* genes across the 93 metagenomes

Supplementary Table 9 Corrected mean coverage of the non-redundant TARA Oceans *nifH* genes and all sequences from three reference collections (the FunGene database, the ‘Zehr database’, and the amplicon sequences, see Material and Methods section) that recruited any read across the 93 metagenomes, and blast results of *nifH* queries (see Methods).

Supplementary Table 10 Genomic features of 30,244 bins manually characterized from the 12 metagenomic sets. Completion and redundancy estimates are based on the average of four bacterial single-copy gene collections.

Supplementary Table 11 KEGG annotation for 1,077 MAGs.

Supplementary Table 12 Relative distribution of 1,077 MAGs across the 93 metagenomes.
