# Author Correction: Nitrogen-fixing populations of Planctomycetes and Proteobacteria are abundant in surface ocean metagenomes

**DOI:** 10.1038/s41564-018-0209-4

**Published:** 2018-07-24

**Authors:** Tom O. Delmont, Christopher Quince, Alon Shaiber, Özcan C. Esen, Sonny TM Lee, Michael S. Rappé, Sandra L. McLellan, Sebastian Lücker, A. Murat Eren

**Affiliations:** 1 Department of Medicine, University of Chicago, Chicago, IL USA; 2 Warwick Medical School, University of Warwick, Coventry, UK; 3 Graduate Program in the Biophysical Sciences, University of Chicago, Chicago, IL USA; 4 Hawaii Institute of Marine Biology, University of Hawaii at Manoa, Kaneohe, HI USA; 5 School of Freshwater Sciences, University of Wisconsin-Milwaukee, Milwaukee, WI USA; 6 Department of Microbiology, Radboud University, Nijmegen, The Netherlands; 7 Josephine Bay Paul Center, Marine Biological Laboratory, Woods Hole, MA USA; 8 Committee on Microbiology, University of Chicago, Chicago, IL USA

**Keywords:** Microbial ecology, Metagenomics, Marine microbiology, Bacterial genomics

Correction to: *Nature Microbiology* 10.1038/s41564-018-0176-9, published online 11 June 2018.

In the version of this Article originally published, the surname of author Sandra L. McLellan was spelt incorrectly as ‘MacLellan’. Table 1 also contained errors: in the ‘Population' column, ‘HBD' was incorrectly spelt as ‘HDB'; in the ‘Taxonomy' column, the family was given as ‘Desulfobacteraceae' for populations HBD-03, HBD-04 and HBD-05 but it should have been ‘Oceanospirillaceae'. These errors have now been corrected in the online versions. In addition, Fig. 2 was also included in the Supplementary Information as Supplementary Fig. 2; this has now been replaced with the correct supplementary figure (shown below).

**Figure 1:**
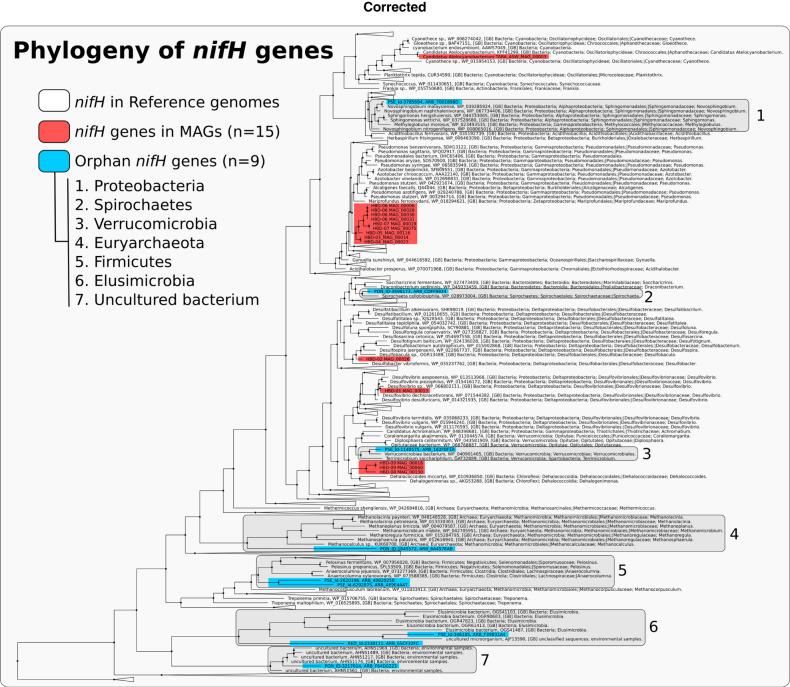
**Supplementary Fig. 2**

