# Supplementary material for: Nitrogen-fixing populations of Planctomycetes and Proteobacteria are abundant in surface ocean metagenomes
Source: Nat Microbiol. 2018 Jun 11;3(7):804–13. doi: 10.1038/s41564-018-0176-9 (PMC6792437; doi:10.1038/s41564-018-0176-9)
Supplement: Supplementary file 1 — Supplementary Notes, Supplementary References, Supplementary Figures 1 and 2, Supplementary Table References. [file 41564_2018_176_MOESM1_ESM.pdf]

SUPPLEMENTARY INFORMATION
https://doi.org/10.1038/s41564-018-0176-9

nature microbiology

In the format provided by the authors and unedited.

Nitrogen-fixing populations of Planctomycetes and Proteobacteria are abundant in surface ocean metagenomes

Tom O. Delmont[1*], Christopher Quince[2], Alon Shaiber[3], Özcan C. Esen[1], Sonny TM Lee[1], Michael S. Rappé[4], Sandra L. McLellan[5], Sebastian Lücker[6] and A. Murat Eren[1,7,8*]

[1]Department of Medicine, University of Chicago, Chicago, IL, USA. [2]Warwick Medical School, University of Warwick, Coventry, UK. [3]Graduate Program in the Biophysical Sciences, University of Chicago, Chicago, IL, USA. [4]Hawaii Institute of Marine Biology, University of Hawaii at Manoa, Kaneohe, HI, USA. [5]School of Freshwater Sciences, University of Wisconsin-Milwaukee, Milwaukee, WI, USA. [6]Department of Microbiology, Radboud University, Nijmegen, The Netherlands. [7]Josephine Bay Paul Center, Marine Biological Laboratory, Woods Hole, MA, USA. [8]Committee on Microbiology, University of Chicago, Chicago, IL, USA. *e-mail: tomodelmont@gmail.com; meren@uchicago.edu

© 2018 Macmillan Publishers Limited, part of Springer Nature. All rights reserved.

Supplementary Information for “Nitrogen-fixing populations of Planctomycetes and Proteobacteria are abundant in the surface ocean” by Delmont et al.

Functional differences between HBDs. Functional differences between HBDs included multiple traits within the biological nitrogen cycle. For instance, hydrolysis of urea to ammonia and CO_2 was characteristic to the gammaproteobacterial HBDs, while oxidation of glycine to ammonia was only detected in the deltaproteobacterial HBDs and the Planctomycetes HBD-08 (Table S6). These pathways might be alternative ammonia sources to avoid costly nitrogen fixation. Similar to the previously observed co-occurrence of nitrogen-fixing and denitrification genes in isolates from coastal waters[1], we detected the complete denitrification pathway in all three genomic replicates of HBD-06, suggesting that widespread HBDs may also be involved in nitrogen loss in the surface ocean. However, we identified this pathway only in HBD-06 and a non-diazotroph MAG (ION 00025) affiliated to the genus *Labrenzia*. The scarcity of nitrogen fixation (10/957) and denitrification (2/957) pathways in our genomic database implies that the metabolic potential of HBD-06 is highly singular for the open ocean. Other functional differences between HBDs included genes related to the regulation of nitrogen fixation. Single copies of the genes encoding *nifX*, *nifQ*, *nifO*, *nifW* and *nifT* were characteristic to Oceanospirillales and Pseudomonadales HBDs, while the two-component nitrogen fixation transcriptional regulator FixJ was only detected in the Planctomycetes HBDs. We also identified a relatively small set of 271 functions (4,608 genes) common to the nine HBDs (Figure 2, panel C). Besides various housekeeping genes and the full gene set

for nitrogen fixation, they encoded chemotaxis and flagellar proteins, transporters for ammonia, phosphate and molybdenum (required for nitrogen fixation), and multiple nitrogen regulatory proteins (Table S6). They also included 477 genes coding for transcriptional regulators. HBDs also shared complete pathways for biosynthesis of acyl-CoA and acetyl-CoA (beta-oxidation and pyruvate oxidation), glycolysis, ABC-2 type transport systems, and a two-component regulatory system for phosphate starvation response (Table S5).

Nitrogen fixation gene synteny. All the catalytic and biosynthetic genes for nitrogen fixation were located in a single operon in HBD-02 and HBD-09. In the three Oceanospirillaceae HBDs as well as HBD-06, *nifE* and *nifN* were co-located distantly from the operon containing both *nifHDK* and *nifB*. Two genes coding for nitrogen regulatory proteins were located in between *nifH* and *nifDK* for the two deltaproteobacterial HBDs only. HBD-07 and HBD-08 were too fragmented to properly infer gene organisations. Overall, nitrogen fixation genes were highly segregated in the HBD genomes, but their organization slightly differed between taxonomical lineages.

Translating genome-wide quantitative read recruitment into cells per liter. we assumed (1) the percentage of reads a population genome recruits from a metagenome translate to the proportion of its cells in the corresponding sample, (2) and 0.5 billion archaeal and bacterial cells occur in each liter of the surface ocean, as previously estimated[2]. Estimating the number of cells from population-level metagenomic read recruitment experiments has caveats: variations in genome sizes can yield over-estimated cell numbers for populations with relatively large genomes,

and the occurrence of pico-eukaryotic genomes can lead to an under-estimation of archaeal and bacterial abundances. Given these limitations, the approach provides an approximation to link observations from environmental metagenomics and qPCR surveys.

REFERENCES

1. Bentzon-Tilia, M., Severin, I., Hansen, L. H. & Riemann, L. Genomics and ecophysiology of heterotrophic nitrogen-fixing bacteria isolated from estuarine surface water. *MBio* **6,** (2015).
2. Whitman, W. B., Coleman, D. C. & Wiebe, W. J. Prokaryotes: the unseen majority. *Proc. Natl. Acad. Sci. U. S. A.* **95,** 6578–6583 (1998).

SUPPLEMENTARY FIGURES

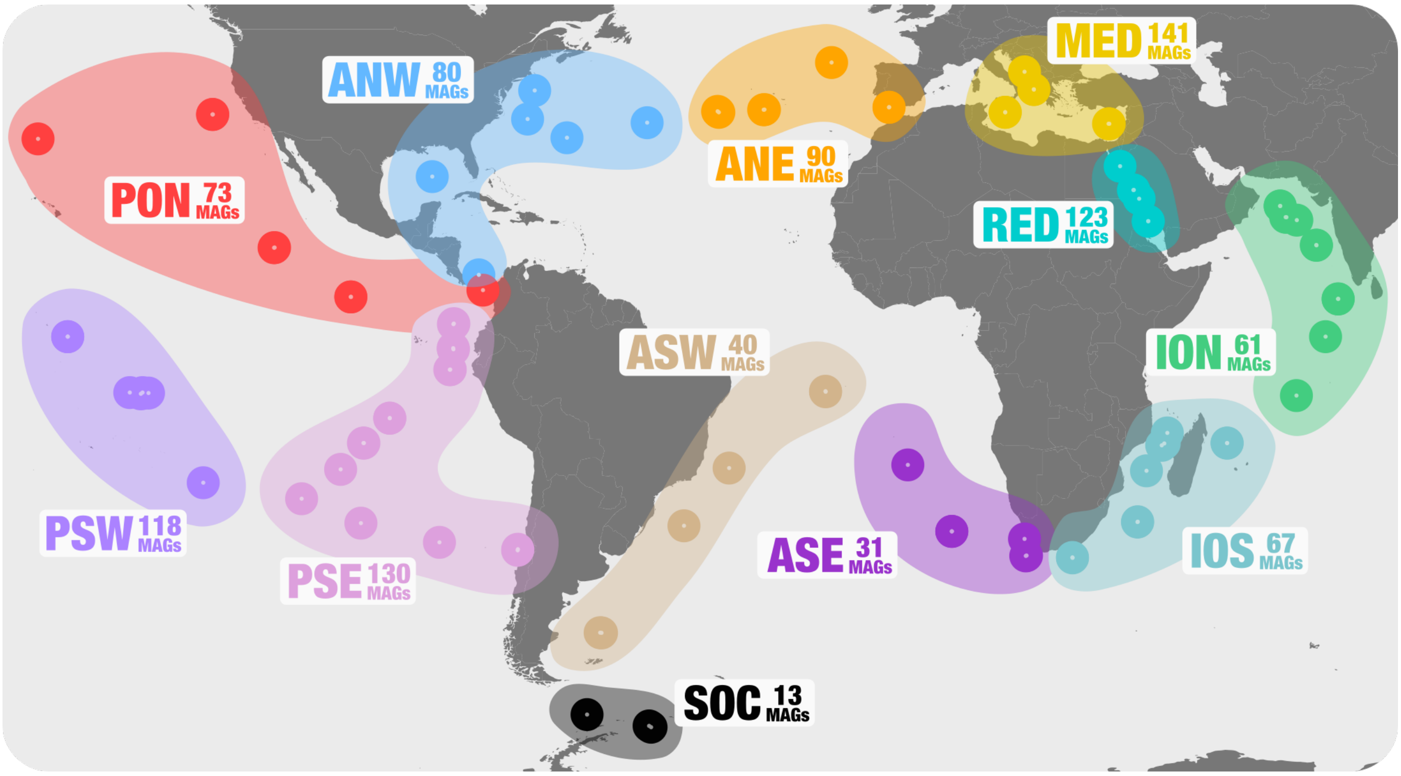

Supplementary Figure 1: Geographically bounded metagenomic co-assemblies. Dots in the map correspond to the geographic location of 93 metagenomes from the TARA Oceans project. Each dot is associated with a metagenomic set (corresponding to a geographic region) for which we performed a metagenomic co-assembly (n=12), These geographic regions include ANE (Atlantic northeast), ANW (Atlantic northwest), ASE (Atlantic southeast), ASW (Atlantic southwest), ION (Indian Ocean north), IOS (Indian Ocean south), MED (Mediterranean Sea), PON (Pacific Ocean north), PSE (Pacific Ocean southeast), PSW (Pacific Ocean southwest), RED (Red Sea), and SOC (Southern Ocean). The figure also displays the number of MAGs recovered from each region.

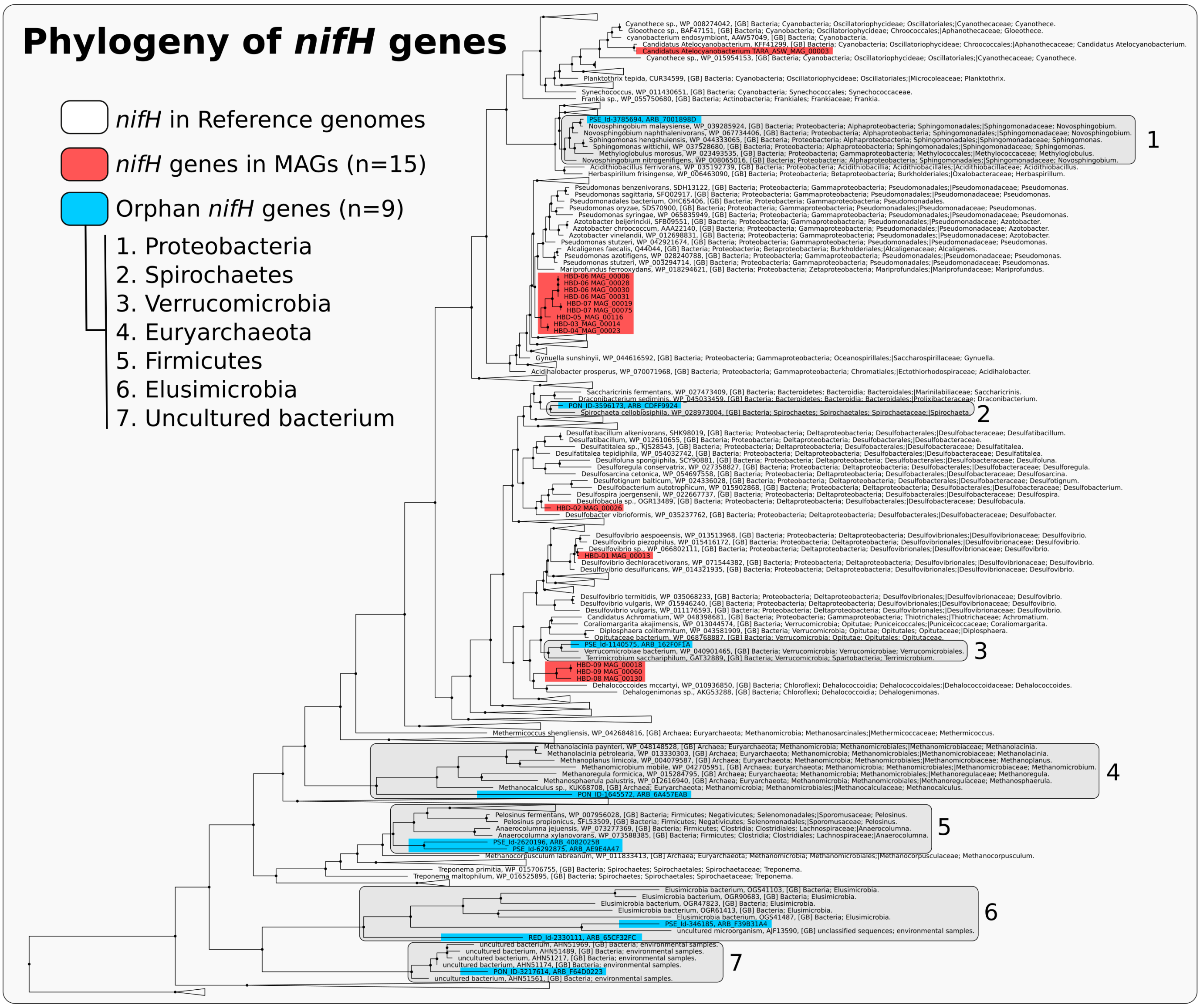

Supplementary Figure 2: Phylogenetic analysis of NifH. The figure describes the phylogenetic affiliation of translated *nifH* genes in 15 nitrogen fixing MAGs (including five redundant MAGs and *Ca*. A. thallassum) and nine orphan scaffolds we identified in this study, as well as 504 reference sequences.

SUPPLEMENTARY TABLES

doi:10.6084/m9.figshare.4902938 gives access to all supplementary tables and figures.

Supplementary Table 1: Summary of the 93 metagenomes from TARA Oceans, and the twelve geographic regions they represent.

Supplementary Table 2: Summary of the co-assembly and binning outputs for each metagenomic set.

Supplementary Table 3: Genomic features of 957 MAGs from the non-redundant genomic database. A two-sided t-test was performed to compare the relative distribution of each MAG in the Pacific Ocean compared to all other locales.

Supplementary Table 4: The 16S rRNA gene sequence identified in HBD-09.

Supplementary Table 5: Genomic features, Pearson correlation (based on the relative distribution in 93 metagenomes) and average nucleotide identity of 1,077 MAGs from the redundant genomic database.

Supplementary Table 6: RAST subsystems and KEGG modules for the nine HBDs.

Supplementary Table 7: Digital droplet PCR assays targeting the *nifH* genes of HBD-08 and HBD-09 (phylum Planctomycetes) in DNA samples from Station ALOHA in the Pacific Ocean. The table lists the newly designed primers and summarizes detection levels in copies per litre.

Supplementary Table 8: Main characteristics of 18 *nifH* genes retrieved in this study, the similarity of reads they recruited across 93 TARA Oceans metagenomes, best matches against the NCBI non-redundant database, *nifH* reference databases and amplicon sequences from a large-scale survey, and compatibility with commonly used PCR primers. The table lists nucleotide sequences of the TARA Oceans *nifH*

genes found in MAGs and orphan scaffolds, and mean coverage of the TARA Oceans *nifH* genes across the 93 metagenomes.

Supplementary Table 9: Corrected mean coverage of the non-redundant TARA Oceans *nifH* genes and all sequences from three reference collections (the FunGene database, the 'Zehr database', and the amplicon sequences, see Material and Methods section) that recruited any read across the 93 metagenomes, and blast results of *nifH* queries (see Methods).

Supplementary Table 10: Genomic features of 30,244 bins manually characterized from the 12 metagenomic sets. Completion and redundancy estimates are based on the average of four bacterial single-copy gene collections.

Supplementary Table 11: KEGG annotation for 1,077 MAGs.

Supplementary Table 12: Relative distribution of 1,077 MAGs across the 93 metagenomes.
