# Supplementary material for: Nitrogen-fixing populations of Planctomycetes and Proteobacteria are abundant in surface ocean metagenomes
Source: Nat Microbiol. 2018 Jun 11;3(7):804–13. doi: 10.1038/s41564-018-0176-9 (PMC6792437; doi:10.1038/s41564-018-0176-9)
Supplement: Supplementary file 2 — Reporting Summary [file 41564_2018_176_MOESM2_ESM.pdf]

natureresearch

Corresponding Author: A. Murat Eren
Date: May 2, 2018

Life Sciences Reporting Summary

Nature Research wishes to improve the reproducibility of the work we publish. This form is published with all life science papers and is intended to promote consistency and transparency in reporting. All life sciences submissions use this form; while some list items might not apply to an individual manuscript, all fields must be completed for clarity.

For further information on the points included in this form, see Reporting Life Sciences Research. For further information on Nature Research policies, including our data availability policy, see Authors & Referees and the Editorial Policy Checklist.

Experimental design

1. Sample size

Describe how sample size was determined.	We used all publically available metagenomic samples from the TARA Oceans project that corresponded to the size fraction of interest.

2. Data exclusions

Describe any data exclusions.	No data was excluded from the analysis.

3. Replication

Describe whether the experimental findings were reliably reproduced.	Experimental replication was not attempted.

4. Randomization

Describe how samples/organisms/participants were allocated into experimental groups.	Metagenomic samples were grouped based on geographical origin.

5. Blinding

Describe whether the investigators were blinded to group allocation during data collection and/or analysis.	Not applicable because the study does not involve animals and/or human research participants.

Note: all studies involving animals and/or human research participants must disclose whether blinding and randomization were used.

6. Statistical parameters

For all figures and tables that use statistical methods, confirm that the following items are present in relevant figure legends (or the Methods section if additional space is needed).

n/a	Confirmed	
☐	☒	The exact sample size (*n*) for each experimental group/condition, given as a discrete number and unit of measurement (animals, litters, cultures, etc.)
☐	☒	A description of how samples were collected, noting whether measurements were taken from distinct samples or whether the same sample was measured repeatedly.
☐	☒	A statement indicating how many times each experiment was replicated
☐	☒	The statistical test(s) used and whether they are one- or two-sided (note: only common tests should be described solely by name; more complex techniques should be described in the Methods section)
☐	☒	A description of any assumptions or corrections, such as an adjustment for multiple comparisons
☐	☒	The test results (e.g. *p* values) given as exact values whenever possible and with confidence intervals noted
☐	☒	A summary of the descriptive statistics, including central tendency (e.g. median, mean) and variation (e.g. standard deviation, interquartile range)
☒	☐	Clearly defined error bars

See the web collection on statistics for biologists for further resources and guidance.

Software

Policy information about availability of computer code

7. Software

Describe the software used to analyze the data in this study.	Most of our analysis was performed using the open-source platform anvi'o (version 2.3.0). The code base is available at https://github.com/merenlab/

anvio. Other software used to analyze the data include MEGAHIT v1.0.3, Prodigal v2.6.3, HMMER v3.1b2, Centrifuge, Bowtie2 v2.0.5, CONCOCT, CheckM, KEGG, RAST, NUCmer, R, ggplot2, PhyloSift, DIAMOND, blastx, ARB v.5.5,
In addition, custom codes used in the study are available from the URL http://merenlab.org/data/2017_Delmont_et_al_HBDs/.

For all studies, we encourage code deposition in a community repository (e.g. GitHub). Authors must make computer code available to editors and reviewers upon request. The *Nature Methods* guidance for providing algorithms and software for publication may be useful for any submission.

Materials and reagents

Policy information about availability of materials

8. Materials availability

Indicate whether there are restrictions on availability of unique materials or if these materials are only available for distribution by a for-profit company.	No unique material was used.

9. Antibodies

Describe the antibodies used and how they were validated for use in the system under study (i.e. assay and species).	No antibodies were used.

10. Eukaryotic cell lines

a. State the source of each eukaryotic cell line used.	No eukaryotic cell lines were used.
b. Describe the method of cell line authentication used.	No eukaryotic cell lines were used.
c. Report whether the cell lines were tested for mycoplasma contamination.	No eukaryotic cell lines were used.
d. If any of the cell lines used in the paper are listed in the database of commonly misidentified cell lines maintained by ICLAC, provide a scientific rationale for their use.	No eukaryotic cell lines were used.

Animals and human research participants

Policy information about studies involving animals; when reporting animal research, follow the ARRIVE guidelines

11. Description of research animals

Provide details on animals and/or animal-derived materials used in the study.	No animals were used.

Policy information about studies involving human research participants

12. Description of human research participants

Describe the covariate-relevant population characteristics of the human research participants.	The study did not involve human participants.
